# Integrated transcriptomic and immunological profiling reveals new diagnostic and prognostic models for cutaneous melanoma

**DOI:** 10.3389/fphar.2024.1389550

**Published:** 2024-05-28

**Authors:** Changchang Li, Nanhui Wu, Xiaoqiong Lin, Qiaochu Zhou, Mingyuan Xu

**Affiliations:** ^1^ Department of Dermatology, Wenzhou Hospital of Integrated Traditional Chinese and Western Medicine, Wenzhou, China; ^2^ Department of Dermatopathology, Shanghai Skin Disease Hospital, Tongji University School of Medicine, Shanghai, China

**Keywords:** SKCM, melanoma, LASSO machine learning algorithm, differential gene expression, immune infiltration analysis

## Abstract

The mortality rate associated with cutaneous melanoma (SKCM) remains alarmingly high, highlighting the urgent need for a deeper understanding of its molecular underpinnings. In our study, we leveraged bulk transcriptome sequencing data from the SKCM cohort available in public databases such as TCGA and GEO. We utilized distinct datasets for training and validation purposes and also incorporated mutation and clinical data from TCGA, along with single-cell sequencing data from GEO. Through dimensionality reduction, we annotated cell subtypes within the single-cell data and analyzed the expression of tumor-related pathways across these subtypes. We identified differentially expressed genes (DEGs) in the training set, which were further refined using the Least Absolute Shrinkage and Selection Operator (LASSO) machine learning algorithm, employing tenfold cross-validation. This enabled the construction of a prognostic model, whose diagnostic efficacy we subsequently validated. We conducted Gene Ontology (GO) and Kyoto Encyclopedia of Genes and Genomes (KEGG) analyses on the DEGs, and performed immunological profiling on two risk groups to elucidate the relationship between model genes and the immune responses relevant to SKCM diagnosis, treatment, and prognosis. We also knocked down the GMR6 expression level in the melanoma cells and verified its effect on cancer through multiple experiments. The results indicate that the GMR6 gene plays a role in promoting the proliferation, invasion, and migration of cancer cells in human melanoma. Our findings offer novel insights and a theoretical framework that could enhance prognosis, treatment, and drug development strategies for SKCM, potentially leading to more precise therapeutic interventions.

## 1 Introduction

SKCM is globally recognized as the third most prevalent type of skin cancer and the 19th most common cancer overall (Davey et al., 2021). Despite constituting only about 1% of all skin cancers, SKCM is the most invasive and perilous type, responsible for 90% of skin cancer-related deaths (Eddy and Chen, 2020). In the United States in 2023, approximately 97,610 new cases of SKCM were projected, accompanied by an estimated mortality of 7,990 (Siegel et al., 2023). *In situ* SKCM represents the precursor stage to malignant SKCM, with its incidence rising even faster than that of malignant SKCM (Wei et al., 2016; Sacchetto et al., 2018). The 5-year survival rate for localized SKCM is 99%, but it drops to 63% for regional metastatic SKCM and further plummets to 20% for distant metastatic cases (Fakhoury et al., 2024). Major risk factors for SKCM include environmental factors like excessive UV exposure, genetic factors, gender, age, race, and immune factors (Hawkes et al., 2016; Davey et al., 2021). Females have a lower risk of SKCM, and their prognosis is better than males (El Sharouni et al., 2019). Socioeconomic status is closely linked to SKCM incidence, with higher socioeconomic status correlating to higher malignant SKCM rates, though tumors are thinner, and prognosis is relatively better compared to lower socioeconomic status patients (Gibson et al., 2020).Despite advancements in surgery, radiation therapy, chemotherapy, and targeted treatments such as KIT inhibitors, SKCM poses challenges due to difficulties in early non-invasive identification, high invasiveness, and early occurrence of local or distant metastases, leading to an overall poor prognosis (Lo and Fisher, 2014; Slipicevic and Herlyn, 2015; Davis et al., 2019). Therefore, in-depth research into the mechanisms of SKCM occurrence and development, especially those leading to metastasis and recurrence, is crucial. Identifying key biomarkers and exploring crucial target genes are essential for the diagnosis, treatment, and prognosis of SKCM.

Numerous studies have investigated the role of specific gene families in SKCM and constructed prognostic models (Luo et al., 2023; Yue et al., 2023). However, these studies have primarily focused on subsequent analyses based on specific functional gene sets. Analyzing the intrinsic correlations and potential therapeutic targets of gene expression from a holistic transcriptomic perspective can provide a more comprehensive understanding of the disease. Additionally, the analysis paradigm has expanded from simple prognostic model construction and molecular mechanism analysis to drug prediction. This “treatment-prognosis” comprehensive analysis offers a theoretical basis for improving cancer treatment. However, existing studies are based on the “pRRophetic” R package (Li AA. et al., 2022; Zhao et al., 2023). Considering the early release year of the “pRRophetic” R package (Geeleher et al., 2014), it is necessary to perform drug prediction for SKCM based on the new “oncopredict” package (Maeser et al., 2021).

In this study, we performed a comprehensive analysis of both bulk transcriptomic and single-cell sequencing data for SKCM, sourced from the public databases of The Cancer Genome Atlas (TCGA) and The Gene Expression Omnibus (GEO). Initially, we applied dimensionality reduction techniques to the single-cell dataset, annotating cellular subtypes and examining the expression of tumor-related pathways across these subtypes. We then leveraged the bulk transcriptomic data to construct predictive models, which were rigorously validated using designated training and validation sets. Differential expression analysis identified a set of genes (DEGs) from the training dataset. These DEGs were further scrutinized using the Least Absolute Shrinkage and Selection Operator (LASSO) machine learning algorithm, enhanced with tenfold cross-validation, to refine the model development and perform validation tests. In addition, we conducted Gene Ontology (GO) and Kyoto Encyclopedia of Genes and Genomes (KEGG) enrichment analyses, as well as mutation analysis on these DEGs. Furthermore, we utilized three immune infiltration algorithms to explore immune-related dynamics within two defined risk groups, uncovering potential links between the prognostic model and tumor immunity. Sensitivity analysis was also employed to guide targeted drug selection. Our findings provide significant new insights and a solid theoretical foundation for advancing the prognosis and therapeutic strategies for SKCM.

## 2 Materials and methods

### 2.1 Data acquisition and preprocessing

We acquired bulk transcriptome sequencing data, mutation data, and clinical information for a melanoma cohort of 457 patients from The Cancer Genome Atlas (TCGA, https://portal.gdc.cancer.gov/), designated as TCGA-SKCM. Additionally, we downloaded bulk transcriptome sequencing data (GSE65904) for 208 melanoma patients and single-cell sequencing data (GSE72056) from The Gene Expression Omnibus (GEO, https://www.ncbi.nlm.nih.gov/geo/). All data utilized in this study are freely available through these public databases, which ensures compliance with ethical standards and eliminates the need for additional ethical approval. Our data acquisition and analysis processes conformed to all relevant guidelines and regulations.

### 2.2 Single-cell sequencing analysis

We analyzed single-cell sequencing data using the “Seurat” package. Our initial steps included stringent quality control and data cleaning to ensure the integrity and accuracy of our analyses. The quality thresholds set were: mitochondrial gene content (percent.mt) less than 10%, a minimum of 1000 RNA counts (nCount_RNA), and RNA feature numbers (nFeature_RNA) between 100 and 5000. Following data preprocessing, we utilized Uniform Manifold Approximation and Projection (UMAP) for dimensionality reduction of the single-cell data. Cell subtypes were then annotated using specific markers for each subtype, with expression distributions visualized through dot plots, violin plots, and feature plots. Additionally, the “cellchat” package facilitated the analysis of intercellular communication, visualizing interactions and quantifying proportions of each cell subtype. To assess pathway activity, the “PROGENy” package calculated pathway scores for each tumor-related pathway in individual cells, averaging these scores to determine the overall pathway activity level for each cell subtype. A heatmap was then generated to display and compare pathway activity levels across 14 tumor-related pathways among different cell subtypes, aiming to elucidate variations in pathway engagement.

### 2.3 Construction and validation of a machine learning prognostic model

The analysis is based on a cohort of 457 melanoma patients from TCGA-SKCM dataset. Differential gene expression analysis was conducted using the “tinyarray” package, with the criteria for selecting DEGs set at *p* < 0.05 and | log^2^(FoldChange) |> 1. Subsequently, DEGs underwent univariate COX regression analysis with a significance threshold of *p* < 0.05 to identify genes influencing prognosis. The identified prognostic genes were subjected to LASSO machine learning algorithm using the “glmnet” and “survival” packages, with ten-fold cross-validation. The LASSO algorithm, along with cross-validation, was employed for further gene selection, and coefficients were calculated to construct the prognostic model. The computation of the risk score for each patient involved multiplying the expression value of each gene by its corresponding coefficient and summing the outcomes. The formula for the risk score is as follows:

$$\text{Risk Score}=\sum_{i=1}^{n}\left[{Expression\;value}_{{gene}_{i}}*{Coefficient}_{{gene}_{i}}\right]$$

In the context provided, the term “Expression value” represents the expression level obtained from the sequencing or chip data of the model genes, while “Coefficient” represents the coefficient corresponding to the model genes when the error is minimized during cross-validation calculations. Individual patient risk scores are calculated, and depending on whether the score exceeds or falls below the median value of all patient risk scores, individuals are categorized into either the high-risk group or the low-risk group. To validate the universality of the model, we selected GSE65904 as an external validation cohort. Risk scores were calculated based on the above formula and method, and patients were grouped accordingly. Validation was performed alongside the training cohort. Risk cumulative factor plots were visually represented for both the training and validation cohorts. Survival curves were also plotted to verify the overall survival (OS) differences between high-risk and low-risk patient groups. Following this, the training cohort underwent univariate COX regression analysis for the selected model genes to ascertain their potential as prognostic factors. Forest plots were generated for visualization. Furthermore, age, gender, and risk score were collectively subjected to univariate COX regression analysis to evaluate their potential as prognostic factors and to compare the magnitude of risk associated with each. We further analyzed the differential expression of model genes between the two risk groups. The expression correlation among model genes was also analyzed and presented using a heatmap. Following this, gender, age, risk score, and metastasis status were incorporated to construct a nomogram prognostic model.

### 2.4 Functional enrichment and mutation analysis

Prior to constructing the LASSO machine learning model, we conducted univariate COX regression analysis on DEGs. Subsequently, we performed Gene Ontology (GO) and Kyoto Encyclopedia of Genes and Genomes (KEGG) analyses to observe the enrichment of these genes in specific functional pathways, visualizing the results through bubble plots. The GO and KEGG analyses were executed using the R package “clusterProfiler” (version 4.0.5), with a significance threshold set at a False Discovery Rate (FDR) < 0.05.

Utilizing the R package “maftools” (version 2.12.0), we analyzed and visualized the differential mutation profiles of DEGs in two risk groups. The waterfall plot presented the top 6 genes in each group, accompanied by statistics on the proportion of nucleotide transitions and transversions. Moreover, our emphasis was on scrutinizing the mutation sites and types of genes exhibiting the highest mutation frequencies among the two risk groups. Employing the “RCircos” package, we generated a circular chromosome plot to annotate the positions of model genes on the chromosomes.

### 2.5 Immune-related analysis

We employed the Spearman correlation method to analyze the relationship between the risk score and 43 immune checkpoint genes, presenting the results in a bar chart. Furthermore, correlations between model genes and the immune checkpoint genes were visualized using a heatmap. To assess immune cell infiltration in two risk groups, we utilized three algorithms: Microenvironment Cell Populations-counter (MCPcounter), Single-sample Gene Set Enrichment Analysis (ssGSEA), and Estimation of Cell Types in Bulk Expression Data (xCell). These algorithms analyzed transcriptome-wide gene expression data to estimate immune cell infiltration scores. Initially, ssGSEA assigned scores to 23 immune cell types for individual patients, with variations depicted in box plots. We then used Spearman correlation to evaluate the relationships between the risk score, model genes, and immune cell levels, visualizing these relationships through scatter plots and heatmaps. The MCPcounter algorithm identified differences in 10 immune cell types between high-risk and low-risk groups, with results shown in box plots and correlations with model genes and risk score illustrated via scatter plots and heatmaps. Utilizing the xCell package, the xCell algorithm computed infiltration scores for 67 immune cell types, with differences between risk groups depicted in box plots and correlations presented in a heatmap.

### 2.6 Drug sensitivity analysis

We accessed drug-related data from the Genomics of Drug Sensitivity in Cancer (GDSC, https://www.cancerrxgene.org/) database using the “oncoPredict” R package. Initial analyses explored differences in drug sensitivity between the two risk groups, visualized through a volcano plot. Spearman correlation was then applied to ascertain the relationships between model genes and 61 drugs, with the findings displayed in a heatmap. Finally, we assessed the variations in drug sensitivity between the risk groups, presenting these findings through box plots.

### 2.7 Cell culture and transfection

In this study, the malignant melanoma cell line of human(A375) was procured from the Cell Bank of the Chinese Academy of Sciences. We cultured the cell line in high-glucose Dulbecco’s Modified Eagle Medium (DMEM, Sigma, Darmstadt, Germany) supplemented with 10% Fetal Bovine Serum (FBS Premium, BI, Israel). Cell culture flasks were maintained in a humidified incubator with 5% CO_2_ at 37°C to promote exponential growth of the cells.

For transfection experiments with the A375 cell line, two primer sequences and one siRNA sequence targeting GRM6 were custom-designed and manufactured by GIMA Corporation, China. Initially, A375 cells were dissociated from culture flasks and resuspended in complete growth medium. Cells were evenly seeded onto 6-well plates at a density of 1 × 104 cells per well, with each well supplemented to a final volume of 2 mL with complete medium. Upon cell adherence, siRNA and the transfection reagent PolyFast (catalog number HY-K1014, MCE, United States) were pre-mixed according to the instructions of manufacturer and incubated at 23°C for 15 min. Then, the mixture was then evenly distributed into the respective wells using a pipette. We replaced culture medium after 6 h of transfection, and subsequent experiments were performed 48 h post-transfection. Primer sequences: GRM6: Forward: 5′- ACT​GAT​CTG​CAG​TGG​CTC​AT - 3′, Reverse: 5′- GCC​CAG​CTT​TGT​GAT​CTT​GT - 3′; β-actin: Forward: 5′- CCTGGCACCCAGCACAAT - 3′, Reverse: 5′- GGG​CCG​GAC​TCG​TCA​TAC- 3′. siRNA: siGRM6: Sense: 5′- ACU​GUU​UAA​GAU​CAG​UAU​A - 3′, Antisense: 5′ -CAA​GTA​TAT​CGC​CTT​CAC​AA - 3′; siNC: Sense: 5′- UUC​UCC​GAA​CGU​GUC​ACG​UTT- 3′, Antisense: 5′ -ACG​UGA​CUC​GUU​CGG​AGA​ATT - 3′.

### 2.8 Total RNA extraction and RT-qPCR

In this study, the RT-qPCR technology was employed to assess the knockdown efficiency of siGRM6. Cells were digested using trypsin (HyClone, United States), followed by three washes with PBS and centrifugation at 4°C to remove the supernatant. Subsequently, 700 μL of Trizol (Takara, Japan) was added to operate lysing procedure on cells according to the manufacturer’s instructions. After incubating on ice for 5 min, 200 μL of chloroform (SINOPHARM, China), 500 μL of isopropanol (SINOPHARM, China), and 1 mL of ethanol (SINOPHARM, China) were added. Before new chemicals were added, full mixing was guaranteed, followed by centrifugation at 4°C and incubation on ice for 15 min. After discarding all organic solvents and air-drying for 20 min, RNA precipitate was obtained.

Then, we added 20 μL of DEPC-treated water to dissolve the precipitate, and we measuring concentration through a Nanodrop 2000 instrument (Thermo, United States). Based on the manufacturer’s instructions, we reverse-transcribed RNA into cDNA using the PrimeScript RT kit (TaKaRa, Japan). Subsequently, we mixed cDNA samples with SYBR GreenER Supermix kit (TaKaRa, Japan). We operated real-time fluorescence quantitative PCR analysis at 7500 Real-Time PCR System (Thermo Fisher Scientific, United States). The parameters of PCR were set according to the SYBR GreenER Supermix kit instructions. Based on the Ct values, the relative expression level on GRM6 was calculated through the method of 2^−ΔΔCT^ normalized to β-actin.

### 2.9 CCK8 assay

After transfection for 48 h, GMR6-NC and GRM6-si cell lines were transferred to a 96-well plate (6000 cells/well) and returned to the incubator for attachment. Three replicate wells were set up for each group. Following the manufacturer’s instructions, CCK8 reagent (KeyGEN, China) was mixed with complete culture medium to ensure a total volume of 200 μL per well, which was swiftly added to the 96-well plate using a pipette. The plate was completely wrapped in aluminum foil to avoid light exposure, and the absorbance at 450 nm for each well was measured on the instrument after 2 h. This process was repeated at 24, 48, 72, and 96-h time points.

### 2.10 EdU staining for DNA replication

“GMR6-NC” and “GRM6-si” cells were seeded at a density of 5 × 104 cells/mL in a 48-well plate and incubated at 37°C for 24 h. Then, 200 μL of EdU culture medium was added to each well, and cells were incubated for 2 h before collection. The 2 cell groups were observed under a fluorescence microscope, and images were taken to record DAPI staining, EdU staining, and merged staining.

### 2.11 Transwell assay

During the study, a layer of matrix gel was coated on the inner surface of the chamber (Thermo, United States) diluted at a ratio of 1:9, with 30 μL added to each chamber. Next, 600 μL of complete culture medium was added to each well of a 24-well plate. After 48 h of transfection, cells were digested and suspended in culture medium without FBS. To ensure the accuracy of the experiment, cells were diluted to a concentration of 30,000 cells per well, with 200 μL of liquid added to each chamber. The chambers were then incubated in the incubator for 24 h. During this period, the liquid in the chambers was removed, and a moist cotton swab was used to wipe off the non-invading cells.

To further analyze the experimental results, the chambers were immersed in polyformaldehyde for 20 min. Subsequently, they were washed three times with PBS and stained with 0.1% crystal violet staining solution for 20 min. After washing again with PBS, the chambers were dried, and images were captured under a microscope for further analysis and discussion.

### 2.12 Wound healing assay

In cell culture experiments, transfected cells were first removed from the culture medium after 48 h, followed by three washes with PBS to clean residual substances. Next, using a 200 μL pipette tip assisted by a ruler, a vertical line was slowly and evenly scratched in each well. To avoid cross-contamination between different wells, the pipette tip was changed with each well. Subsequently, basic culture medium without FBS was added to each well, and the area of the scratch wound at time 0 was observed and photographed under a microscope. The plate was then placed in the cell culture incubator for cultivation, and photographs were taken again after 48 h to record the area of the healed wound. Finally, the percentage of scratch closure was calculated to evaluate the growth and repair ability of the cells.

### 2.13 Statistical analysis

All statistical analyses were conducted using R software (version 4.1.3). COX regression analysis was performed with the “survival” and “survminer” packages. Differential expression analysis utilized the “limma” package, and visualization tasks were predominantly carried out using “ggplot2”. Statistical significance was established at a threshold of *p* < 0.05, with significance levels marked as *p < 0.05, **p < 0.01, ***p < 0.001, and ****p < 0.0001.

## 3 Results

### 3.1 Single-cell sequencing analysis

**FIGURE 1:**
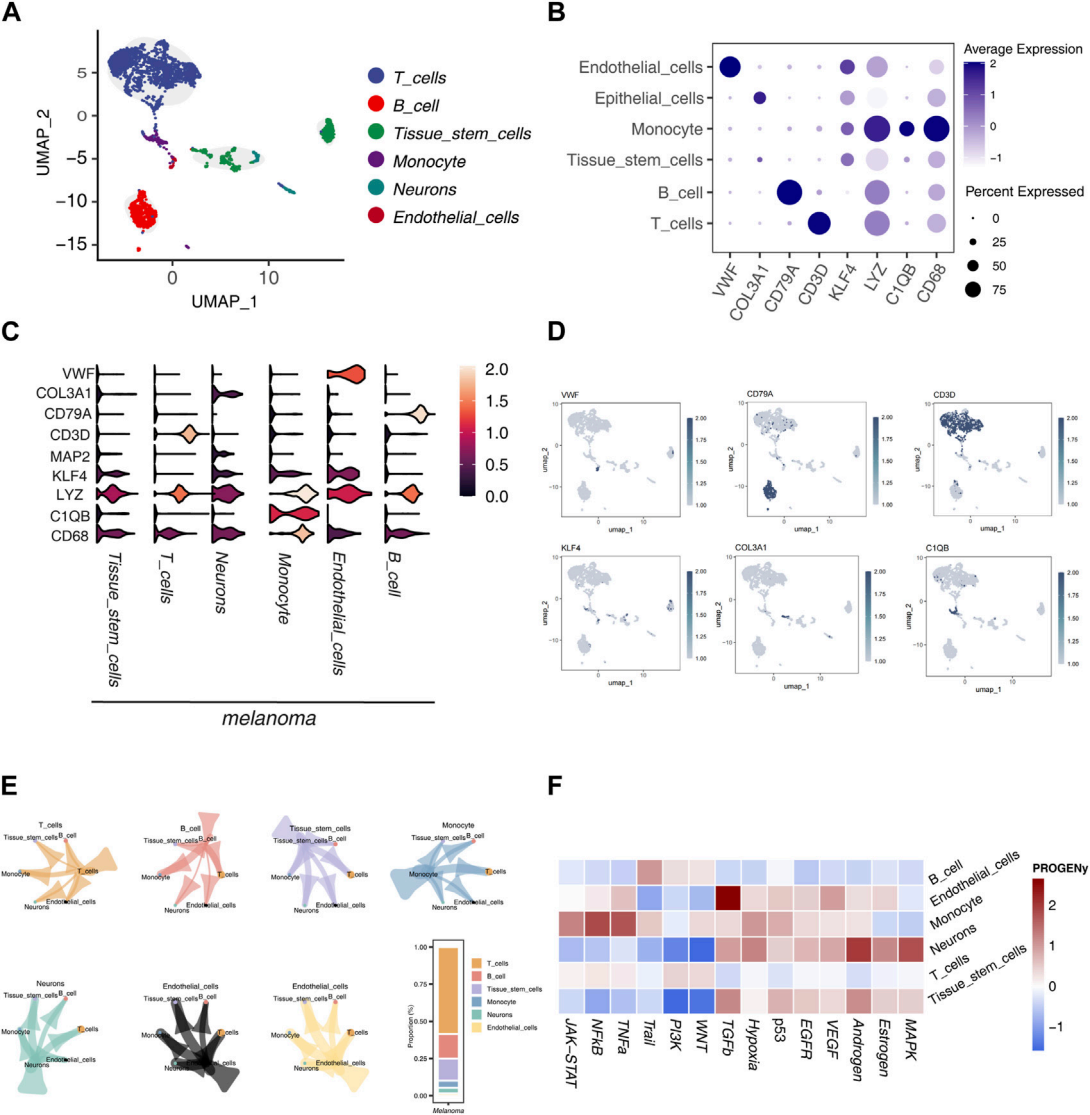
Single-Cell Sequencing Data Analysis **(A)** UMAP dimensionality reduction and annotation of single-cell sequencing data GSE72056, categorizing cell subgroups into 6 classes. **(B)** Dot plot illustrating the differential expression of marker genes in different subgroups. **(C)** Violin plot displaying the differential expression of marker genes in different subgroups. **(D)** Coloring and marking marker gene expression distribution in UMAP visualization. **(E)** Analysis of communication relationships between cell subgroups using the “cellchat” package, visualized. **(F)** Heatmap displaying the score differences of each tumor-related pathway calculated by the “PROGENy” package in each cell.

UMAP dimensionality reduction was applied to single-cell sequencing data (GSE72056), resulting in the classification of cells into six distinct subtypes: T cells, B cells, tissue stem cells, monocytes, neurons, and endothelial cells (Figure 1A). Among these, endothelial cells exhibited the highest expression of von Willebrand factor (VWF), with significant levels of KLF4 and LYZ also noted. In monocytes, LYZ, C1QB, and CD68 demonstrated elevated expression compared to other subtypes and markers. CD79A was the most expressed gene in B cells, whereas CD3D was predominant in T cells (Figure 1B). Violin plots further illustrated these gene expression variations across subtypes.

Notably, CD68 not only showed the highest expression in monocytes but also exceeded the expression of other markers in different cell subtypes. Similarly, LYZ exhibited a widespread expression pattern. KLF4 was more prominently expressed in neurons and monocytes. The neuron subtype showed higher expression of MAP2 and COL3A1 (Figure 1C). Each marker gene’s expression was color-coded in the UMAP visualization to enhance the clarity of their distribution across subtypes (Figure 1D).

We also explored the communication relationships between cell subtypes, revealing extensive signaling interactions. T cells were the most communicative, particularly with other immune cells, followed by B cells (Figure 1E). An analysis of 14 tumor-related pathways indicated differential activation across cell subtypes. B cells showed higher activity levels in the WNT, PI3K, and Trail pathways, although other pathways exhibited lower activity, suggesting a restricted role in tumor-related functions. Endothelial cells displayed the highest activity in the TGF-β pathway, with notable activity in the Estrogen and VEGF pathways, indicating their dynamic involvement in tumorigenesis. Monocytes had elevated activity in the TNF-α, NF-κB, and JAK-STAT pathways, reflecting significant pathway activation. Neurons showed elevated activity in the MAPK, Estrogen, and Androgen pathways but lower activity in the WNT and PI3K pathways, highlighting a selective pathway engagement. T cells exhibited low activity across all pathways examined. Tissue stem cells showed notable activity in the Androgen and TGF-β pathways, with reduced activity in the WNT and PI3K pathways, suggesting a selective activation pattern (Figure 1F).

### 3.2 Constructing and validating of a machine learning prognostic model

**FIGURE 2:**
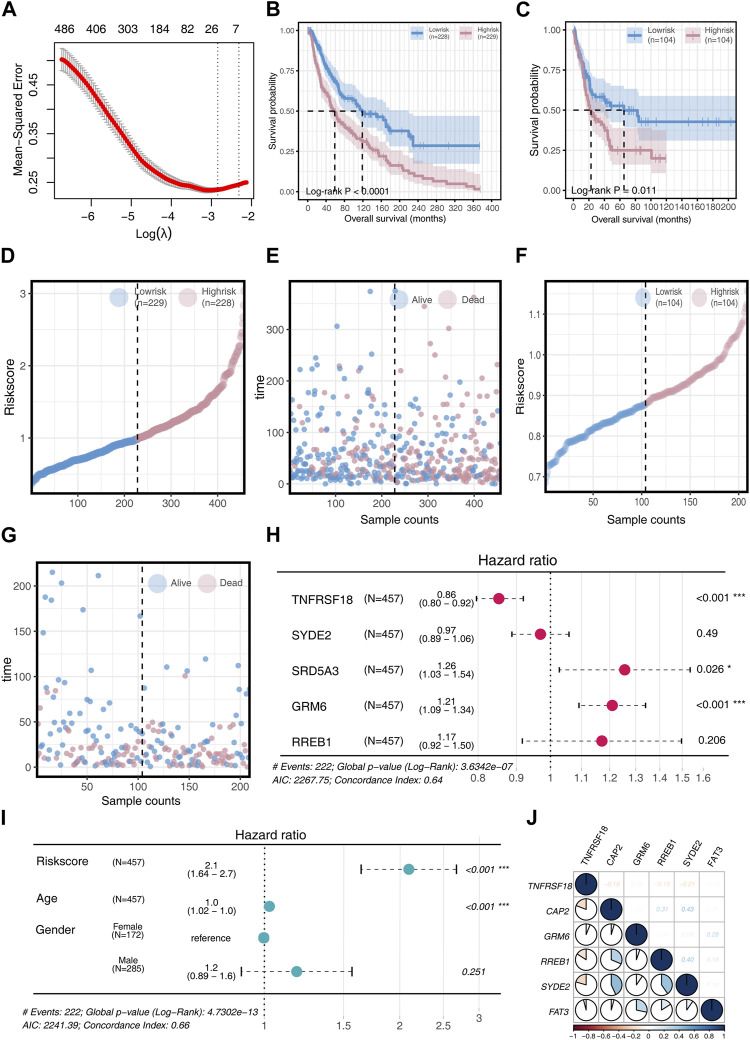
Model Construction and Validation **(A)** Acquisition of DEGs for univariate COX regression analysis and implementation of LASSO machine learning to construct a prognostic model. **(B)** Survival differences in two risk groups in the training set. **(C)** Survival differences in two risk groups in the validation set. **(D)** Risk score changes in two risk groups in the training set. **(E)** Survival time comparison in two risk groups in the training set. **(F)** Risk score changes in two risk groups in the validation set. **(G)** Survival time comparison in two risk groups in the validation set. **(H)** Univariate COX regression analysis to determine if model genes can serve as prognostic factors, visualized through a forest plot. **(I)** Univariate COX regression analysis to determine if Risk score, Age, and Gender can serve as prognostic factors, visualized through a forest plot. **(J)** Analysis of inter-gene correlations in the model.

In our study, the SKCM patient cohort served as the training set, while the GSE65904 patient cohort was used for validation. Initial analysis identified differentially expressed genes (DEGs), and a univariate COX regression analysis was conducted. These DEGs were further refined using a LASSO machine learning algorithm, resulting in a prognostic model comprising six key genes: TNFRSF18, CAP2, GRM6, RREB1, SYDE2, and FAT3, as depicted in Figure 2A. The risk score was computed for each patient using the formula:

Risk score=TNFRSF18*−0.585+CAP2 *0.328+GRM6*0.305+RREB1*1.986+SYDE2 *−0.223+FAT3*−0.006

Patients were categorized into high-risk and low-risk groups based on the median risk score. The high-risk group demonstrated significantly poorer overall survival (OS) than the low-risk group (*p* < 0.05, Figures 2B,C). A cumulative risk factor plot showed an increasing trend of deceased patients and a decline in extended OS with rising risk scores (Figures 2D–G).

Further univariate COX regression analysis highlighted TNFRSF18, SRD5A3, and GRM6 as significant prognostic factors. TNFRSF18 was associated with a protective effect (HR = 0.86), while SRD5A3 (HR = 1.26) and GRM6 (HR = 1.21) were linked to poorer prognosis (Figure 2H). The risk score and age were both significant prognostic factors (*p* < 0.001), with the risk score providing more substantial prognostic information (HR = 2.1) compared to age (HR = 1.0) (Figure 2I).

Correlation analysis among model genes revealed that TNFRSF18 mostly exhibited negative correlations with other genes. Conversely, positive correlations were observed among the remaining model genes, with CAP2 showing strong positive associations with RREB1 (R = 0.31) and SYED2 (R = 0.31), GRM6 with FAT3 (R = 0.28), and RREB1 with SYED2 (R = 0.40) (Figure 2J).

**FIGURE 3:**
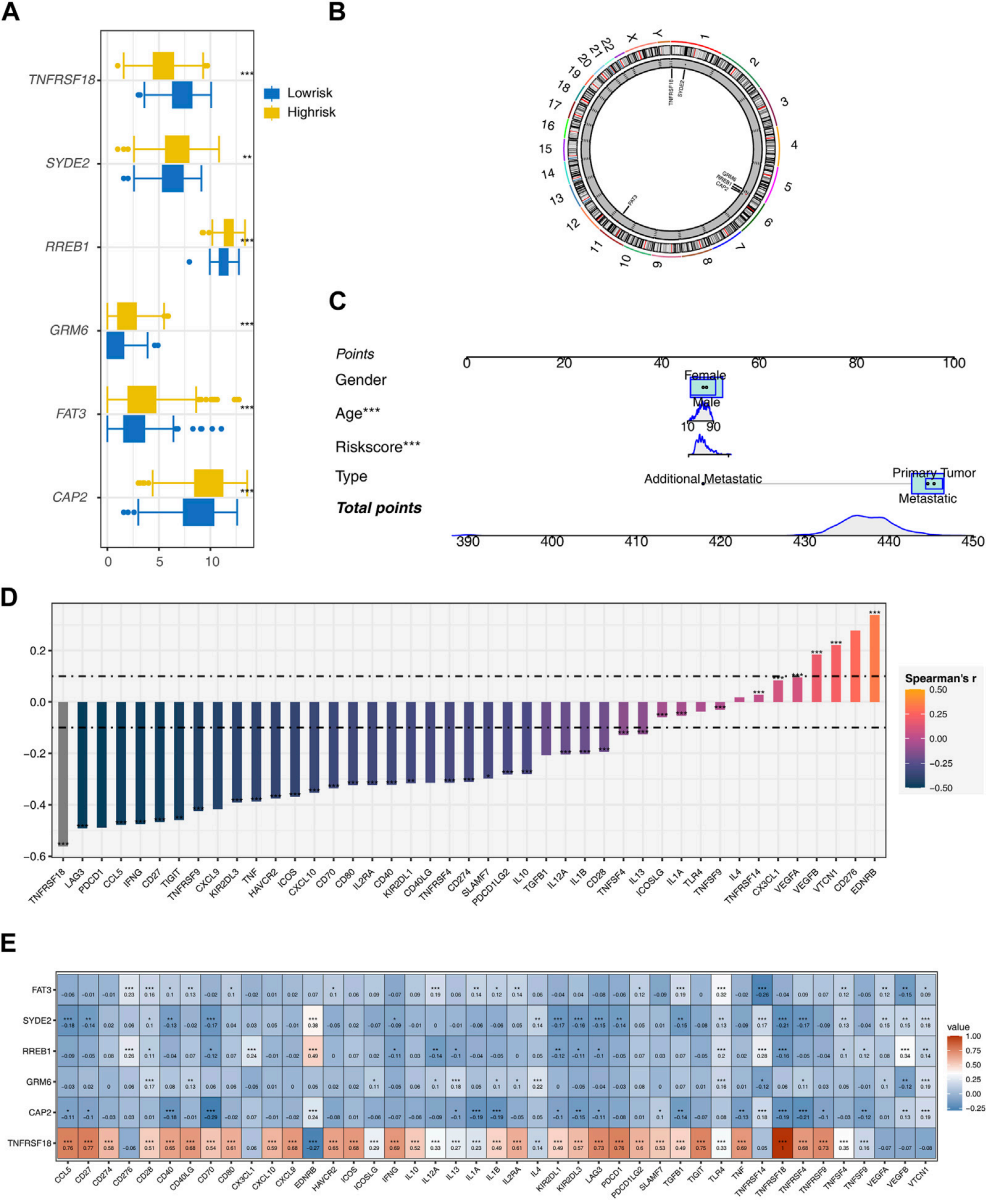
Further Analysis of the Model **(A)** Expression differences of model genes between two risk groups. **(B)** Chromosome circular plot displaying the genomic locations of model genes. **(C)** Construction of a nomogram prognostic model incorporating Risk score, Age, Gender, and Type. **(D)** Calculation of the correlation between the riskscore model and 43 immune checkpoint genes using Spearman’s correlation method. **(E)** Heatmap presenting the expression correlations between model genes and immune checkpoint genes.

Gene expression analyses between the two risk groups showed higher expression levels of all model genes, except for TNFRSF18, in the high-risk group (*p* < 0.01, Figure 3A). Chromosome circle plots highlighted the genomic locations of the model genes (Figure 3B).

A nomogram integrating risk score, age, and type was constructed to enhance the prognostic model’s accuracy (Figure 3C). Spearman correlation analysis identified mostly negative correlations between the risk score and most immune checkpoint genes, except for positive correlations with EDRNB, VTCN1, and VEGFB (Figure 3D). Most model genes also displayed significant negative correlations with immune checkpoint genes, with the notable exception of TNFRSF18, which showed significant positive correlations (Figure 3E).

### 3.3 Enrichment analysis and mutation analysis

**FIGURE 4:**
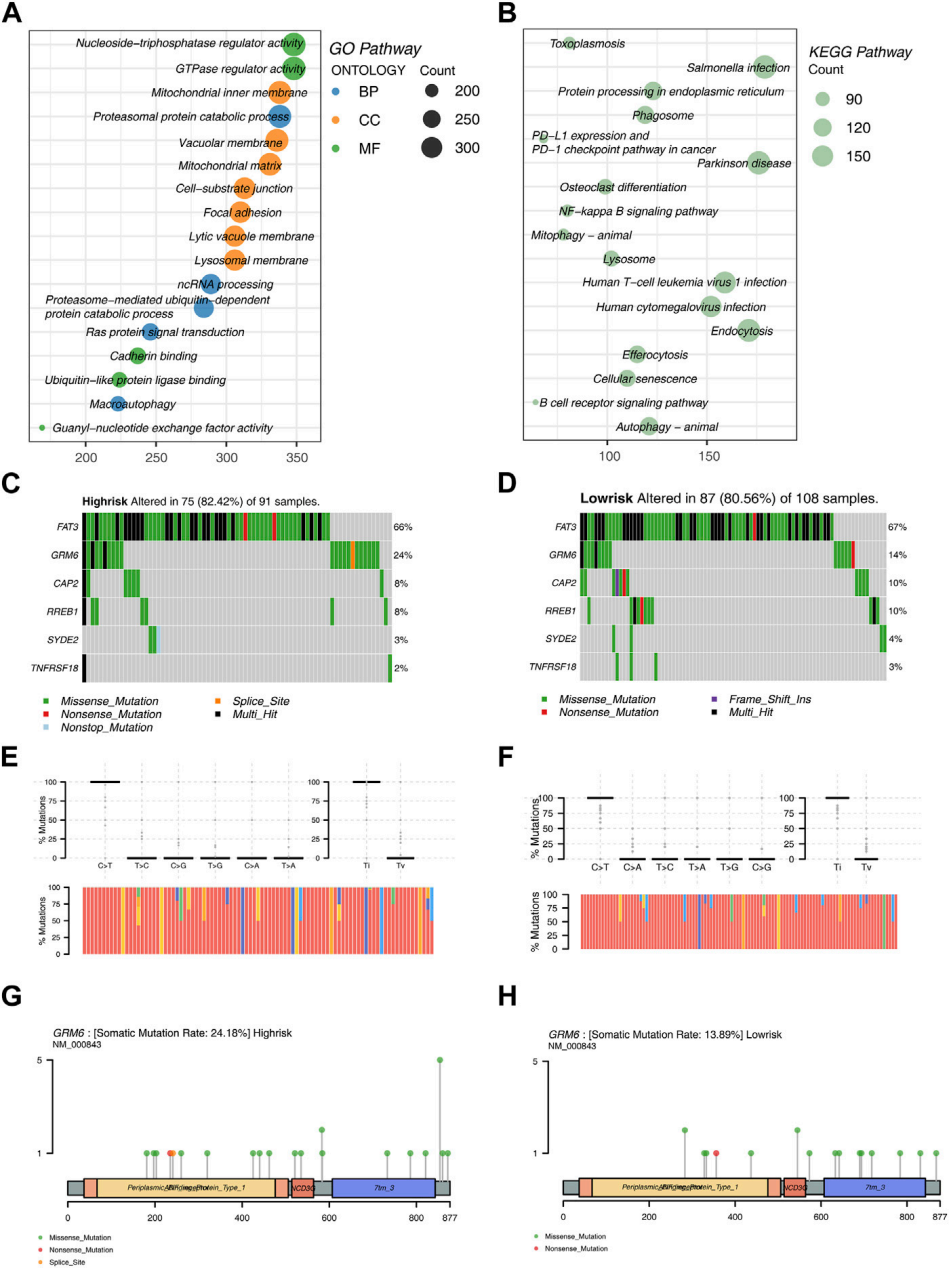
Enrichment Analysis and Mutation Analysis **(A)** Bubble plot illustrating enriched functional pathways in GO analysis of DEGs. **(B)** Bubble plot illustrating enriched functional pathways in KEGG analysis of DEGs. **(C)** Mutation analysis in the high-risk group of the training set. **(D)** Mutation analysis in the low-risk group of the training set. **(E)** Top 6 genes and the proportion of nucleotide transitions and transversions in the high-risk group. **(F)** Top 6 genes and the proportion of nucleotide transitions and transversions in the low-risk group. **(G)** Mutation sites and types of GRM6 in the high-risk group. **(H)** Mutation sites and types of GRM6 in the low-risk group.

Prior to constructing the LASSO machine learning model, we conducted univariate COX regression analysis of DEGs (Differentially Expressed Genes). Following this, GO and KEGG analyses were conducted on the DEGs. The GO analysis revealed that DEGs are predominantly enriched in the number of pathways related to Cell Component (CC). In general, DEGs exhibit predominant enrichment in biological pathways and processes, encompassing energy metabolism, substance metabolism, cell signal transduction, cell structure and dynamics, and protein processing (Figure 4A).

The KEGG analysis results indicated that DEGs are primarily enriched in biological processes such as cell signal transduction, cell metabolism, cell growth and death, and endocytosis (Figure 4B).

Additionally, we conducted mutation analysis on the training dataset. The results showed that FAT3, GRM6, CAP2, RREB1, SYDE2, and TNFRSF18 exhibit higher mutation frequencies in both risk groups. FAT3 has the highest mutation rate in both risk groups, followed by GRM6. Other gene mutation rates are significantly lower compared to these two. In the analysis of mutation types, Missense Mutation appeared most frequently, followed by Multi Hit. FAT3 exhibited various mutation forms, with Missense Mutation and Multi Hit being the most prevalent (Figures 4C,D).

Regarding the analysis of mutation frequencies, the transition (Ti) frequency was higher than the transversion (Tv) frequency in both risk groups. Among them, the nucleotide substitution rate of C>T was the highest (Figures 4E,F).

Moreover, mutation sites and types of GRM6 were analyzed in the two risk groups. Within the high-risk group, GRM6 demonstrated an elevated mutation rate, a wider spectrum of mutation locations, and a greater diversity of mutation types (Figures 4G,H).

### 3.4 Immune-related analysis

**FIGURE 5:**
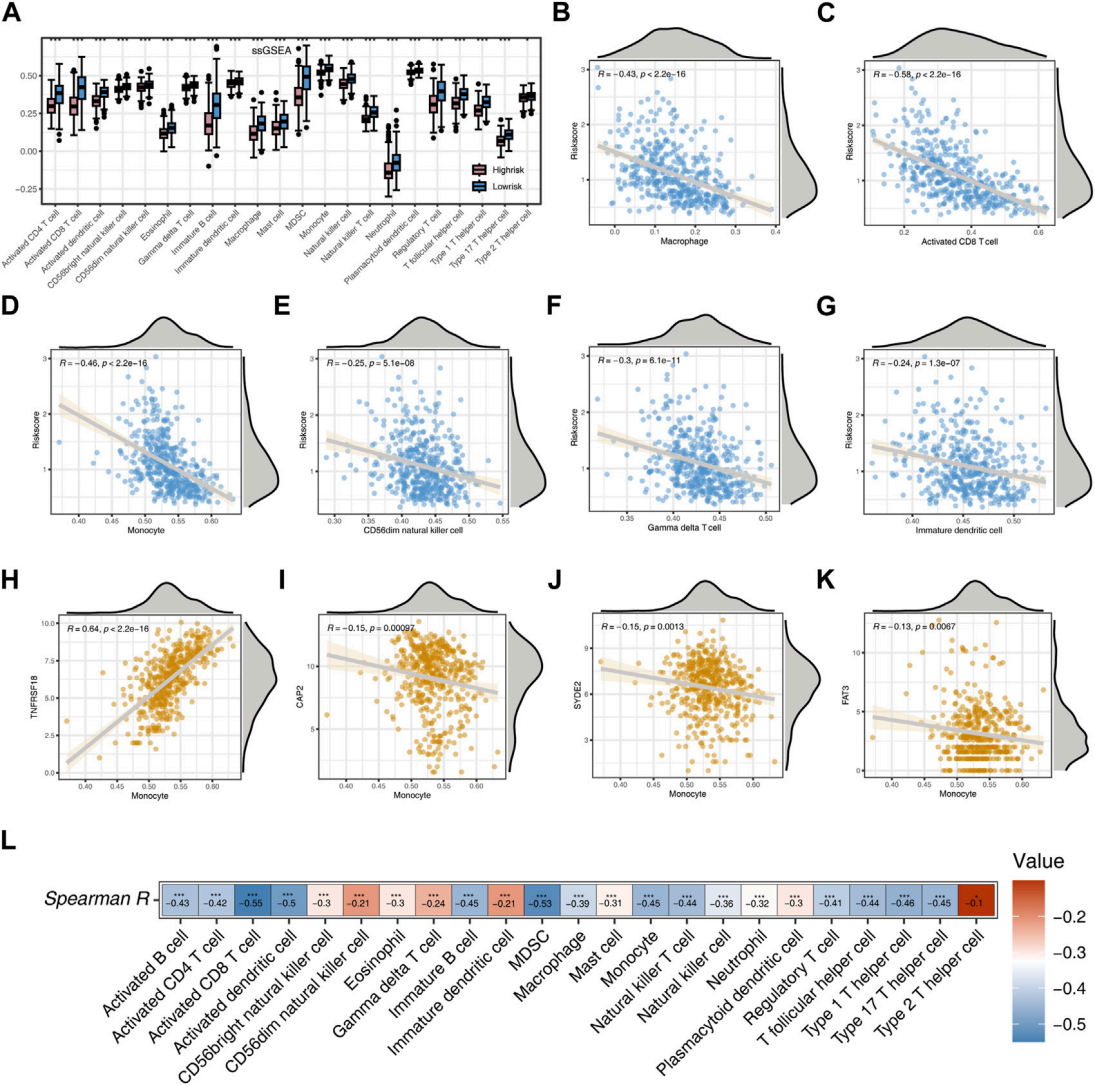
Analysis Based on ssGSEA Immune Algorithm **(A)** Immunocell scoring using the ssGSEA algorithm for two risk groups. **(B)** Correlation between Risk score and Macrophage. **(C)** Correlation between Risk score and Activated CD8 T cell. **(D)** Correlation between Risk score and Monocyte. **(E)** Correlation between Risk score and CD56dim natural killer cell. **(F)** Correlation between Risk score and Gamma delta cell. **(G)** Correlation between Risk score and Immature dendritic cell. **(H)** Correlation between TNFRSF18 and Monocyte. **(I)** Correlation between CAP2 and Monocyte. **(J)** Correlation between SYDE2 and Monocyte. **(K)** Correlation between FAT3 and Monocyte. **(L)** Heatmap of the correlation between risk score and immune cells. Significance levels are denoted as follows: *p < 0.05, **p < 0.01, and ***p < 0.001.

In this study, we employed the ssGSEA algorithm to perform an immune-related analysis, evaluating the infiltration of immune cells in two distinct risk groups. For each patient in the two risk groups, we calculated scores for 23 immune cells. Statistically significant distinctions were noted in the scores of the 23 immune cells between the two risk groups (*p* < 0.05). Remarkably, the scores of all immune cells in the low-risk group surpassed those in the high-risk group, signifying a heightened immune infiltration activity in the low-risk group (Figure 5A).

Subsequent analysis employing Spearman correlation unveiled noteworthy negative associations between the risk score and Macrophage, Activated CD8 T cell, Monocyte, CD56dim natural killer cell, Gamma delta cell, and Immature dendritic cell (*p* < 0.001, R < −0.2, Figures 5B–G). TNFRSF18 demonstrated a marked positive correlation with Monocyte (*p* < 0.001, R = 0.64), whereas CAP2, SYDE2, and FAT3 displayed substantial negative correlations with Monocyte (*p* < 0.001, R < −0.1, Figures 5H–K). Heatmap results further demonstrated a significant negative correlation between the risk score and all immune cells (*p* < 0.05), with the highest negative correlation observed between the risk score and Activated CD8 T cell (R = −0.55), and the lowest negative correlation with Type 2 T helper cell (R = −0.1, Figure 5L).

**FIGURE 6:**
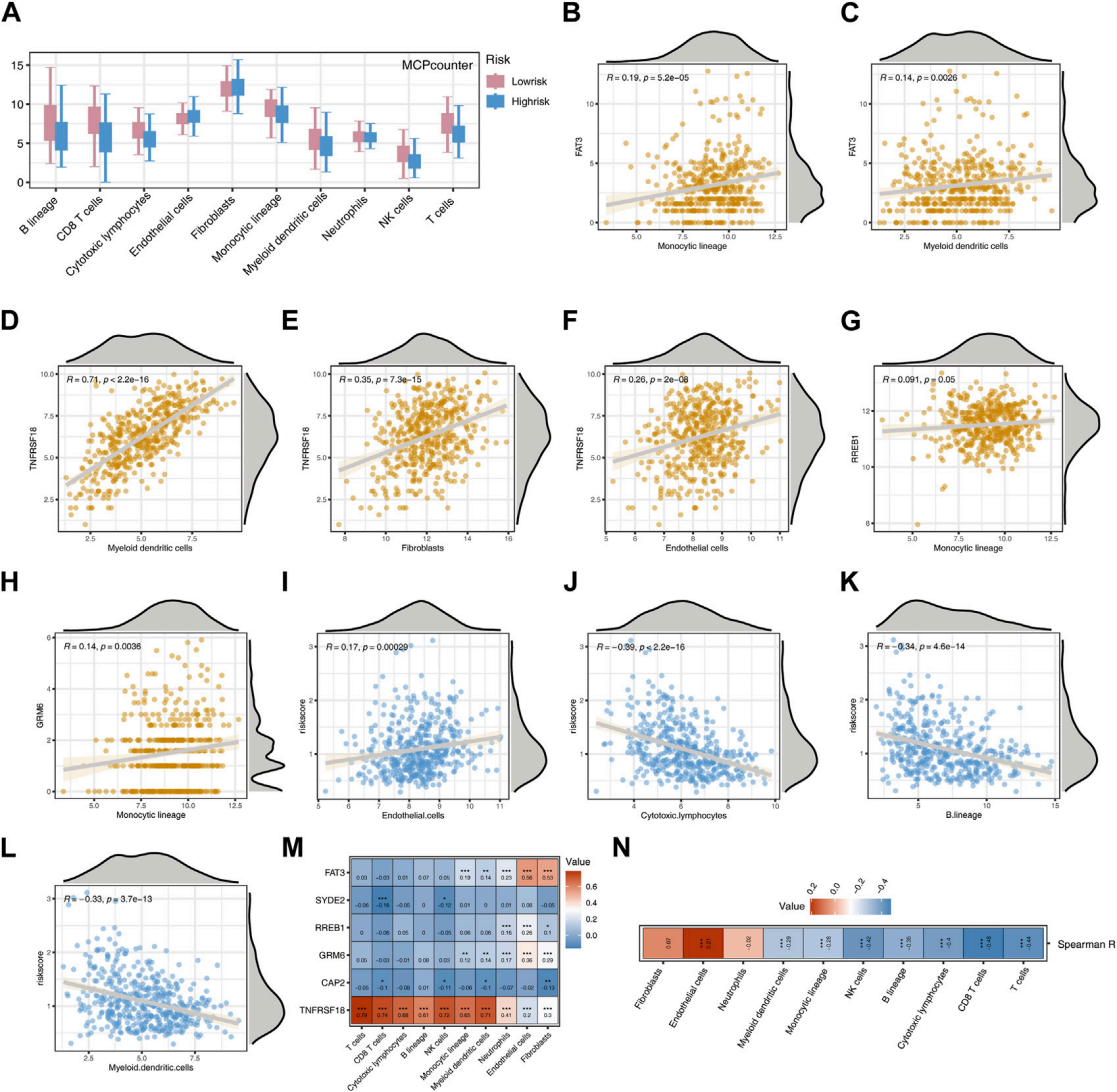
Analysis Based on MCPcounter Immune Algorithm **(A)** Boxplot showing differences in the scores of 10 immune cell types between two risk groups. **(B)** Correlation between FAT3 and Monocyte lineage. **(C)** Correlation between FAT3 and Myeloid dendritic cells. **(D)** Correlation between TNFRSF18 and Myeloid dendritic cells. **(E)** Correlation between TNFRSF18 and Fibroblasts. **(F)** Correlation between TNFRSF18 and Endothelial cells. **(G)** Correlation between RREB1 and Monocyte lineage. **(H)** Correlation between ERM6 and Monocyte lineage. **(I)** Correlation between Endothelial cells and Risk score. **(J)** Correlation between Cytotoxic lymphocytes and Risk score. **(K)** Correlation between B lineage and Risk score. **(L)** Correlation between Myeloid dendritic cells and Risk score. **(M)** Heatmap representing the correlation between model genes and immune cells. **(N)** Heatmap representing the correlation between Risk score and immune cells.

**FIGURE 7:**
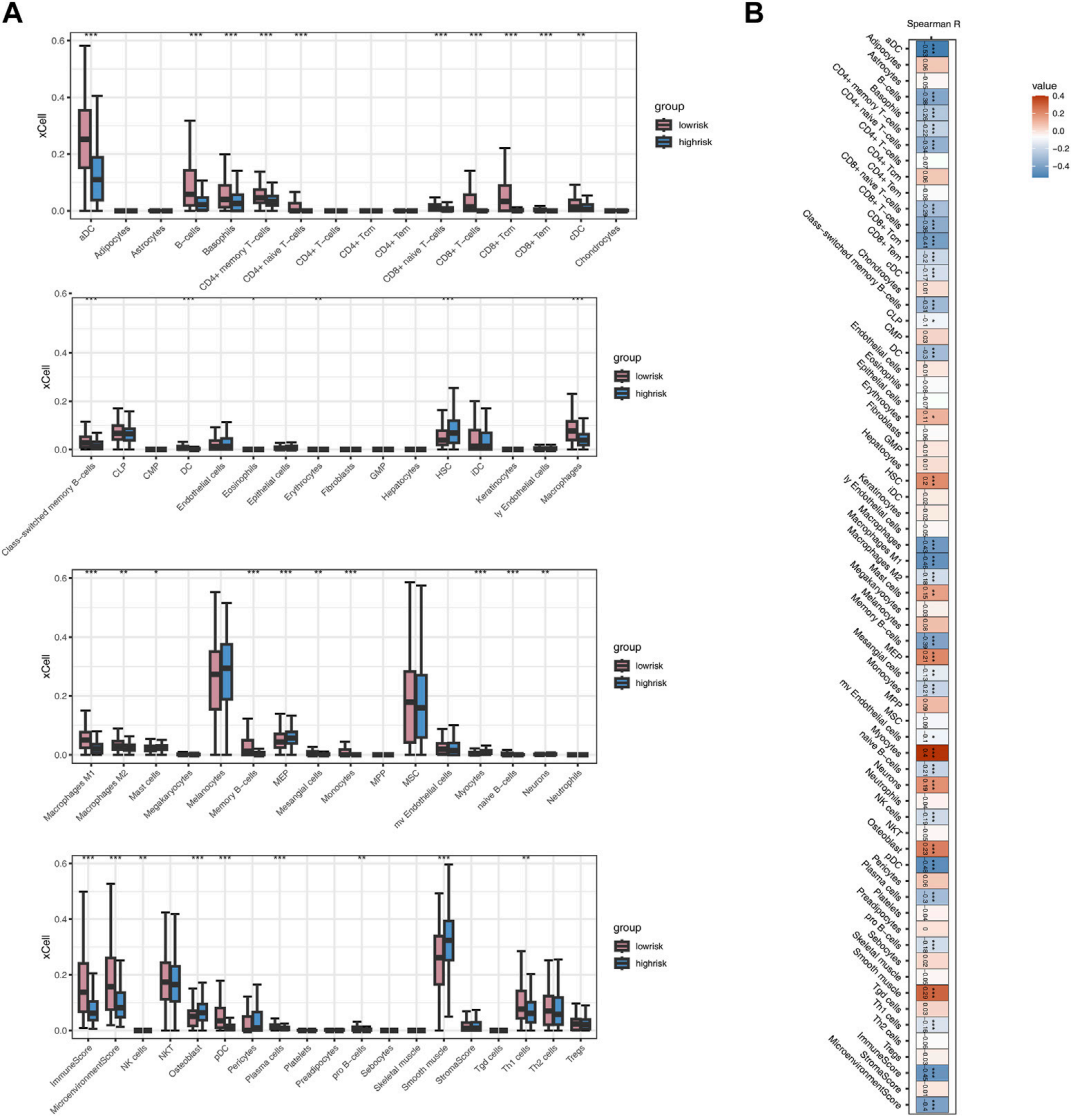
Analysis Based on xCELL Immune Algorithm **(A)** Boxplot illustrating differences in immune cell infiltration between two risk groups. **(B)** Heatmap showing the correlation between Risk score and immune cells.

Employing the MCPcounter algorithm, we computed variations in scores for 10 immune cells between the high-risk and low-risk groups. The findings suggested elevated infiltration levels of the majority of immune cells in the low-risk group, with the exception of Endothelial cells and Fibroblasts (Figure 6A). Spearman correlation analysis suggested positive correlations between most model genes, especially TNFRSF18, and immune cells (*p* < 0.05, R > 0.1, Figures 6B–H). The risk score exhibited a noteworthy positive correlation with Endothelial cells (*p* < 0.001, R = 0.17) and marked negative correlations with Cytotoxic lymphocytes, Myeloid dendritic cells, and B lineage (*p* < 0.001, R < −0.3, Figures 6I–L). The heatmap indicated that, except for TNFRSF18, most model genes were negatively correlated with immune cells, while FAT3 exhibited a highly positive correlation with Endothelial cells and Fibroblasts (*p* < 0.001, R > 0.5, Figure 6M). The risk score demonstrated a significant positive correlation exclusively with Endothelial cells and exhibited negative correlations with the majority of other cells (Figure 6N). The high-risk group shows significantly higher levels of infiltration for several immune cell types compared to the low-risk group (Figure 7A). Additionally, the risk score is negatively correlated with specific immune cells, such as Activated CD8 T cells (Figure 7B).

### 3.5 Drug sensitivity analysis

**FIGURE 8:**
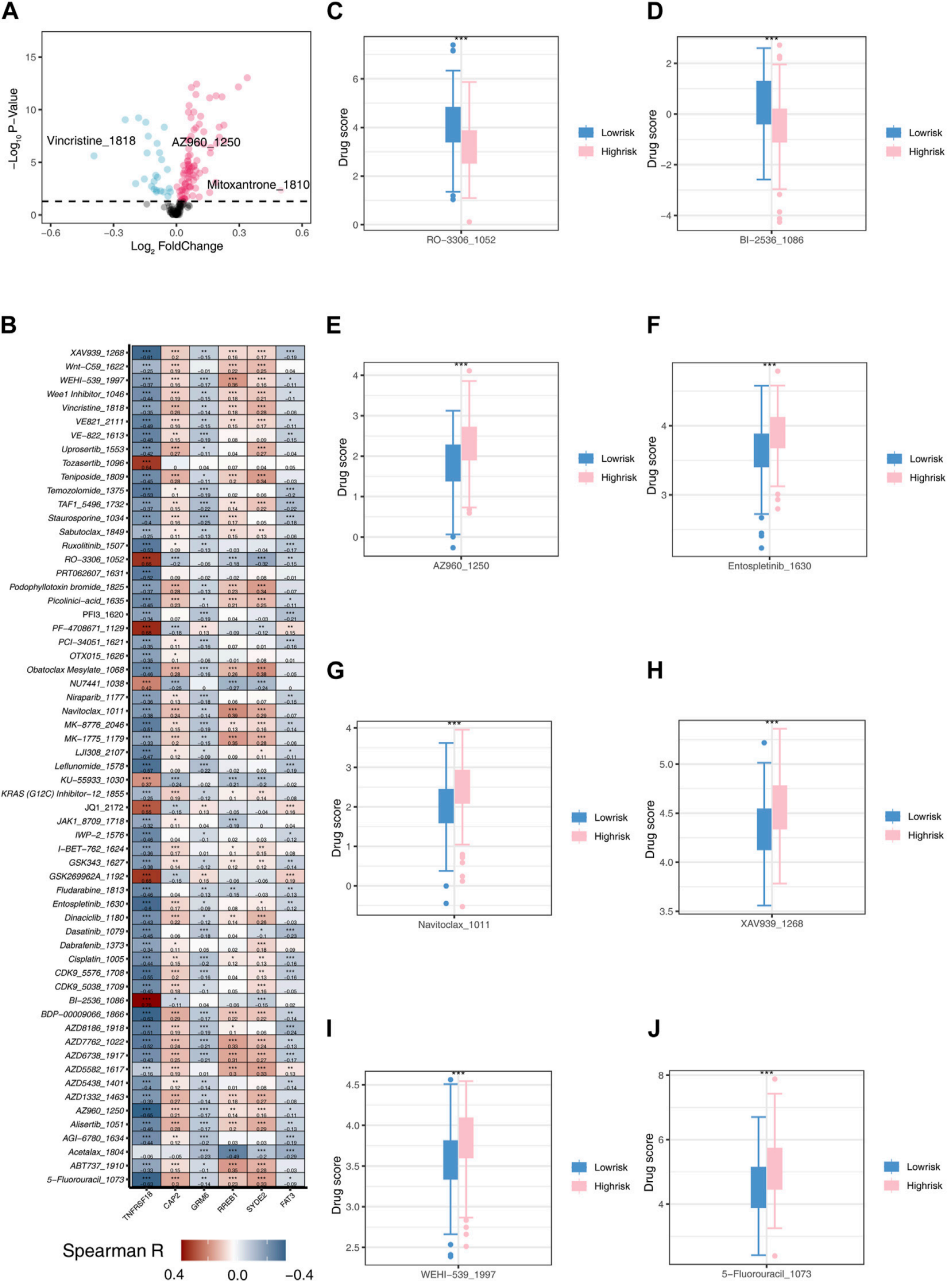
Drug Sensitivity Analysis **(A)** Volcano plot illustrating differences in drug sensitivity between two risk groups. **(B)** Heatmap depicting the correlation between model genes and 61 different drugs. **(C)** Boxplot showing the sensitivity difference of RO-3306_1052 between two risk groups. **(D)** Boxplot showing the sensitivity difference of BI-2536_1086 between two risk groups. **(E)** Boxplot showing the sensitivity difference of AZ960_1250 between two risk groups. **(F)** Boxplot showing the sensitivity difference of Entospletinib_1630 between two risk groups. **(G)** Boxplot showing the sensitivity difference of Navitoclax_1011 between two risk groups. **(H)** Boxplot showing the sensitivity difference of XAV939_1268 between two risk groups. **(I)** Boxplot showing the sensitivity difference of WEHI-539_1997 between two risk groups. **(J)** Boxplot showing the sensitivity difference of 5-Fluorouracil1073 between two risk groups.

We initially performed an analysis of divergent drug sensitivity between the two risk groups and illustrated the outcomes through a volcano plot (Figure 8A). TNFRSF18, GRM6, and FAT3 exhibited a negative correlation with most drugs, while CAP2, RREB1, and SYDE2 showed a positive correlation with most drugs (Figure 8B). RO-3306_1052 and BI-25361086 demonstrated higher drug scores in the low-risk group (Figures 8C,D), whereas AZ960_1250, Entospletinib_1630, Navitoclax1011, XAV939_1268, WEHI-5391997, and 5-Fluorouracil1073 exhibited higher drug scores in the high-risk group (Figures 8E–J). Personalized drug selection for treatment based on individual patient groups may result in improved therapeutic outcomes.

### 3.6 Impact of GRM6 knockdown on A375 melanoma cell functions

**FIGURE 9:**
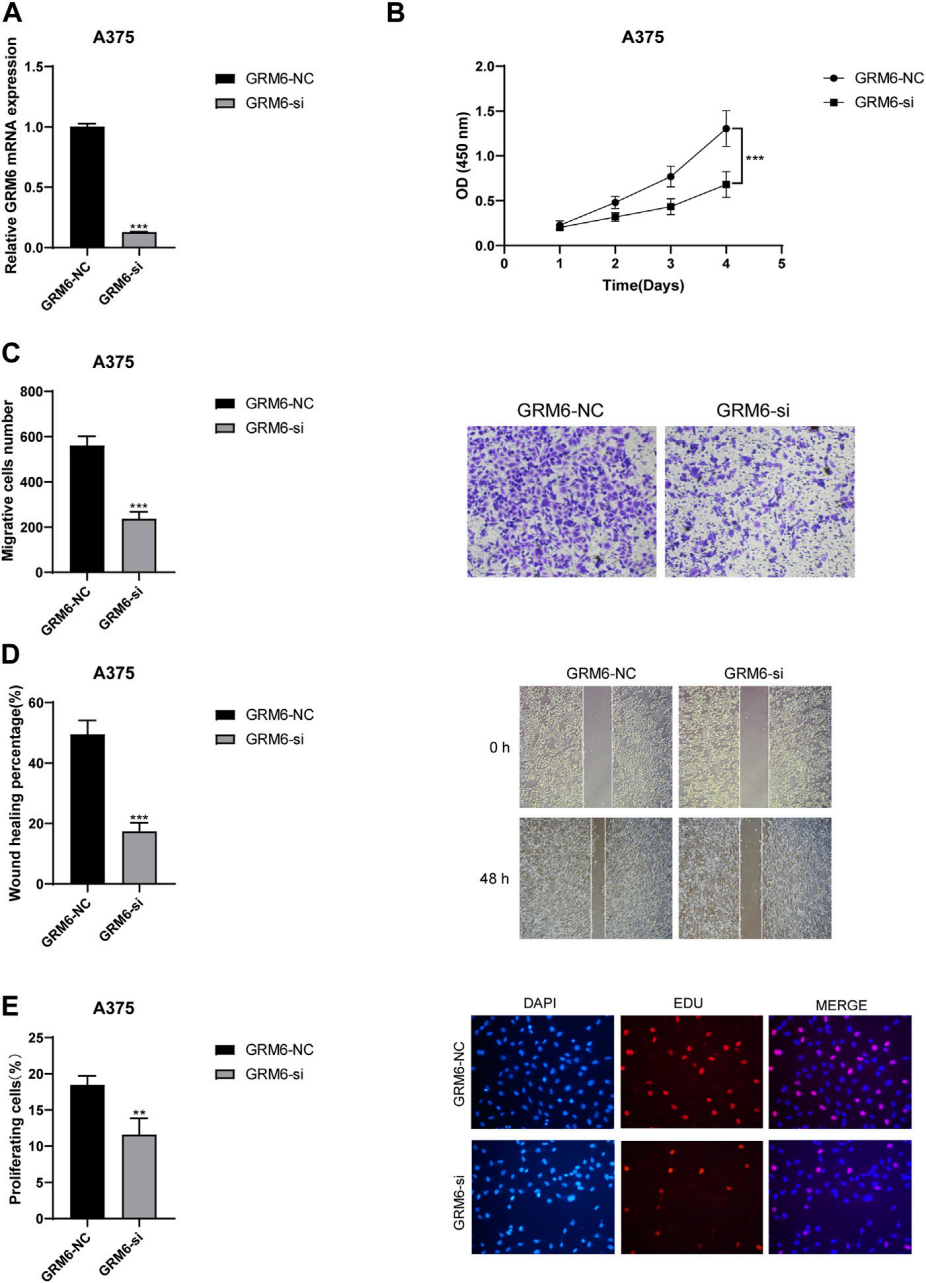
Functional consequences of GRM6 knockdown in A375 melanoma cells. **(A)** RT-qPCR results showing effective knockdown of GRM6 expression in the “GRM6-si” group compared to the control “GRM6-NC” group. **(B)** CCK8 assay results indicating a significant reduction in cell proliferation ability following GRM6 gene silencing. **(C)** Transwell assay results demonstrating decreased invasion ability in A375 cells after GRM6 knockdown. **(D)** Wound healing assay data revealing reduced migration ability of cells in the si-GRM6 group. **(E)** EdU assay results confirming a significant decrease in proliferation rates in GRM6-silenced A375 cells.

Through RT-qPCR experiments, we thoroughly investigated the expression of the “GRM6-NC” control group and the “GRM6-si” knockdown group in the A375 cell line. It was accurately determined that GRM6-si had a good knockdown effect (Figure 9A). CCK8 assays confirmed that the proliferation ability of the A375 cell line significantly decreased when the GRM6 gene was knocked down (Figure 9B). Transwell assays confirmed that after knocking down the GRM6 gene, the number of invasive cells in the si-GRM6 group decreased, which means the invasion ability correspondingly weakened (Figure 9C). We also found that the wound healing assay showed that the migration ability of the si-GRM6 group was significantly reduced (Figure 9D). EdU experiments also confirmed a significant decrease in the proliferation ability of the A375 cell line when the GRM6 gene was knocked down (Figure 9E). Overall, our research results revealed the role of the GRM6 gene in promoting cancer in human melanoma, achieved by promoting the proliferation, invasion, and migration ability of melanoma cells.

## 4 Discussion

Cutaneous melanoma (SKCM) is a type of skin cancer that initially impacts patient quality of life minimally. However, its non-invasive discrimination during early diagnosis is challenging, often resulting in missed opportunities for optimal treatment when patients first seek medical attention. Malignant SKCM is highly invasive, with about 20% of patients experiencing metastasis at the time of initial diagnosis. Advanced-stage malignant SKCM often responds poorly to radiation and chemotherapy, resulting in severe side effects and a grim prognosis. The lack of specific treatments for SKCM, other than early surgical excision, highlights the critical need for research into the mechanistic roles of SKCM-related genes, the construction of prognostic models, and the prediction of drug responses to improve early diagnosis, precise treatment, and patient outcomes.

To investigate the genetic landscape of SKCM, we accessed bulk transcriptome sequencing data from the public databases TCGA and GEO, partitioning these into training and validation sets for robust model construction and validation. Additionally, we collected mutation data and clinical information from TCGA and acquired single-cell sequencing data for SKCM from GEO. These datasets hold significant potential for enhancing patient diagnosis, treatment, and prognosis assessment.

Using the single-cell dataset GSE72056, we applied UMAP dimensionality reduction and annotated cells into six subtypes using specific marker genes for each subgroup. Communication analysis among cell subtypes highlighted active immune cell subgroups, such as T cells and B cells, within SKCM tissue, suggesting a potential limitation in their effectiveness at tumor suppression, possibly due to mechanisms allowing SKCM cells to evade immune surveillance.

Comparison of cell subgroups across 14 tumor-related pathways revealed substantial differences. Both endothelial cells and monocytes showed elevated activity in several pathways, with endothelial cells exhibiting the highest activity in the TGF-β pathway. Given the pivotal role of endothelial cells in angiogenesis, these findings suggest that SKCM may promote angiogenesis via the TGF-β pathway to facilitate nutrient acquisition and metastasis. Additionally, notable pathway activities were observed in neurons and tissue stem cells, with the androgen pathway most active in neurons, raising questions about the role of androgens in the onset and progression of melanoma. Further experimental studies are required to validate these hypotheses.

Utilizing multiple independent datasets from diverse platforms for model construction and validation enhances the model’s generalization capability, leading to more compelling conclusions. This strategy is currently widely employed in the analysis of various diseases (Li J. et al., 2022; Guan et al., 2022). We conducted differential gene expression analysis on patient data from the training set to identify DEGs. Subsequently, we performed univariate COX regression analysis on DEGs to filter out genes that significantly impact prognosis. The selected genes underwent LASSO machine learning algorithm, applying L1 regularization to enhance the model’s simplicity and accuracy by imposing a penalty on the absolute sum of regression coefficients. Ultimately, a prognostic model comprising six genes (TNFRSF18, CAP2, GRM6, RREB1, SYDE2, FAT3) was constructed. TNFRSF18, also known as GITR, is a co-stimulatory T-cell receptor and a member of the TNF receptor superfamily (Nocentini and Riccardi, 2009). Some cancer patients have shown therapeutic efficacy in checkpoint inhibition of TNFRSF18, particularly in preclinical models. However, TNFRSF18 involvement is ineffective in controlling late-stage, immunogenically poor tumors such as B16 SKCM (Hirschhorn et al., 2021).CAP2, a muscle actin-binding protein, regulates cell processes by controlling the dynamics of the cell cytoskeleton (Pelucchi et al., 2023). Its expression in cancerous tissues significantly surpasses that in non-tumor tissues, rendering it a plausible diagnostic and prognostic marker for individuals with cancer (Li et al., 2020).GRM6, also known as mGluR6, is a major excitatory neurotransmitter in the central nervous system. It activates ionotropic and metabotropic glutamate receptors, mediating glutamate synaptic transmission between photoreceptors and ON bipolar cells (Varin et al., 2021). SKCM may lead to SKCM-related retinal lesions, as evidenced in GRM6 (Dhingra et al., 2011).RREB1, a transcription factor that specifically binds to RAS response elements (RRE) on gene promoters, is associated with scrotal SKCM (Thiagalingam et al., 1996; Fujimoto-Nishiyama et al., 1997; Zhang et al., 1999; Date et al., 2004; Mukhopadhyay et al., 2007).SYDE2, an activator of Rho GTPase, has unclear functional implications in tumorigenesis. Studies suggest a potential tumor-suppressive role of SYDE2 in advanced clear cell renal cell carcinoma (Cui et al., 2022).FAT3, a member of the cadherin-related family, has been previously correlated with adverse prognosis in cancer patients (Jiang et al., 2023).

In the training and validation sets, we categorized patients into high-risk and low-risk groups. The survival prognosis of patients in the high-risk group was significantly lower than that in the low-risk group, indicating substantial potential for our model to predict patient outcomes. Among the six genes in the prognosis model, we identified TNFRSF18 as having a protective effect on prognosis. Expression differences were observed for all model genes between the two risk groups, with TNFRSF18 showing higher expression in the low-risk group. In the examination of the correlation with immune checkpoint genes, TNFRSF18 exhibited a significant positive correlation with most immune checkpoint genes. This suggests that TNFRSF18 may serve as an immunotherapeutic target for SKCM, and therapeutic approaches aimed at activating TNFRSF18 expression while inhibiting negative immune regulatory activity may enhance the efficacy of SKCM immunotherapy. Further experimental validation is needed to confirm our hypotheses.

To explore the functional implications of differentially expressed genes (DEGs), we conducted Gene Ontology (GO) and Kyoto Encyclopedia of Genes and Genomes (KEGG) analyses. These DEGs were predominantly enriched in biological processes such as cell signaling, metabolism, growth and death, and endocytosis. We hypothesize that the high activity across these biological pathways contributes to SKCM’s malignancy, highlighting potential avenues for therapeutic intervention.

A mutational analysis performed on the training set identified FAT3 as having the highest mutation rate, closely followed by GRM6. Notably, GRM6 mutations were particularly prevalent within the high-risk group, characterized by a variety of mutation types and locations. These findings underline the need for further comprehensive studies to understand the impact of GRM6 mutations and aberrant expression on melanoma progression.

Immunological correlation analysis, utilizing three distinct immune cell infiltration algorithms, assessed the differences in immune cell infiltration between the two risk groups. The analysis revealed that the low-risk group displayed more active immune infiltration than the high-risk group. Scoring differences between these groups showed that most model genes, particularly TNFRSF18, positively correlated with immune cell activity. Conversely, the risk score exhibited a negative correlation with most immune cells, reinforcing our previous findings that high-risk SKCM correlates with poorer prognosis. This analysis also emphasizes the critical role of TNFRSF18 in the context of SKCM treatment. Additionally, drug sensitivity analysis conducted on the two risk groups highlighted significant differences in their response to various drugs. Based on these findings, we advocate for personalized drug selection strategies tailored to distinct patient subgroups to optimize therapeutic outcomes.

## 5 Conclusion

In conclusion, this study successfully delineated the complex molecular landscape of SKCM, revealing significant findings through the analysis of DEGs, mutation profiles, and immunological correlations. Our comprehensive examination of transcriptomic and mutational data enabled the identification of key genes that are enriched in crucial biological processes and exhibit high mutation rates, such as GRM6 and FAT3, suggesting their pivotal roles in SKCM pathogenesis. The construction and validation of a prognostic model highlighted the differential risk and survival outcomes between patient subgroups, underscoring the importance of early and accurate risk stratification in clinical practice. Moreover, our findings on immune cell infiltration and drug sensitivity emphasize the potential of personalized medicine in treating SKCM, advocating for tailored therapeutic approaches based on individual genetic and immunological profiles. Ultimately, this study provides valuable insights into the underlying mechanisms of SKCM, proposes new therapeutic targets, and supports the advancement of personalized treatment strategies that could significantly improve patient outcomes.

## Data Availability

The datasets analyzed during this study are available on the GEO website (https://www.ncbi.nlm.nih.gov/geo/) and the TCGA website (https://portal.gdc.cancer.gov/). Furthermore, we have uploaded the complete raw data to Nutstore, accessible via this link: https://www.jianguoyun.com/p/DauTKoYQ64nLDBjhys4FIAA.
